# Activator protein-1 (AP-1) inhibition prevents endothelial to mesenchymal transition in diabetes-associated atherosclerosis: a translational study

**DOI:** 10.1186/s12933-025-03060-5

**Published:** 2026-01-29

**Authors:** Abdul Waheed Khan, Misbah Aziz, Karly C Sourris, Jairo P Cortes, Tomasz J Block, Aozhi Dai, Scott Maxwell, Jun Okabe, Emma Pyper, Francesco Paneni, Mark E Cooper, Karin AM Jandeleit-Dahm

**Affiliations:** 1 Department of diabetes, Central Clinical School of Translational Medicine, Monash University, The Alfred Centre, Level 5, 99 Commercial Road, Melbourne, VIC 3004 Australia; 2 Leibniz Institute for Diabetes Research, Heinrich Heine University, Dusseldorf, Germany; 3 Epigenetics in Human Health and Disease Program, Baker Heart and Diabetes Institute, Melbourne, Australia; 4 Center for Translational and Experimental Cardiology (CTEC), Department of Cardiology, University Hospital Zurich and University of Zürich, Wagistrasse 12, Schlieren, 8952 Switzerland

**Keywords:** AP-1 complex, T-5224, EndMT, Diabetes associated atherosclerosis

## Abstract

**Background:**

Endothelial to mesenchymal transition (EndMT), the transformation of endothelial cells into a mesenchymal-like state, is regulated by various factors, including transcription factors such as activator protein 1 (AP-1). While recent studies have confirmed the role of EndMT in atherosclerosis, the involvement of AP-1 in EndMT, particularly in the context of human diabetes, remains unclear.

**Objectives:**

This study aimed to elucidate the role of the AP-1 transcription factor complex in EndMT associated with atherosclerosis in diabetes, utilising both an* in vivo* preclinical model and an ex vivo model using patient-derived serum for translational relevance. Additionally, it sought to profile gene expression changes following AP-1 inhibition in an EndMT model under high glucose conditions.

**Methods:**

Serum from patients with and without type 2 diabetes mellitus (T2DM) was used to assess EndMT in primary human aortic endothelial cells (HAECs) in the presence and absence of the AP-1 inhibitor T-5224. EndMT was evaluated through immunofluorescent staining of these cells and of aortic sections from a murine model of diabetes-associated atherosclerosis in a preclinical early intervention study. Furthermore, HAECs were used to explore the effects of AP-1 inhibition on the transcriptional signature of EndMT.

**Results:**

Patient-derived serum induced EndMT in HAECs, which T-5224 effectively prevented, as confirmed by immunofluorescent staining. Immunofluorescent analysis of the aortic sinus also revealed that T-5224 treatment inhibited EndMT, leading to reduced atherosclerosis in *Apoe*^−/−^ mice. In parallel, in the HAECs-based* in vitro* EndMT model, T-5224 mitigated TNF-α and high glucose-induced EndMT. RNA sequencing identified 242 differentially expressed genes (DEGs) associated with EndMT under high glucose conditions, with T-5224 treatment restoring the expression of 77 DEGs.

**Conclusion:**

This study identifies AP-1 inhibition with T-5224 as a potential therapeutic approach for EndMT resulting in reduced atherosclerosis in diabetes. The use of human serum underscores the translational relevance of these findings.

**Graphical abstract:**

**Supplementary Information:**

The online version contains supplementary material available at 10.1186/s12933-025-03060-5.

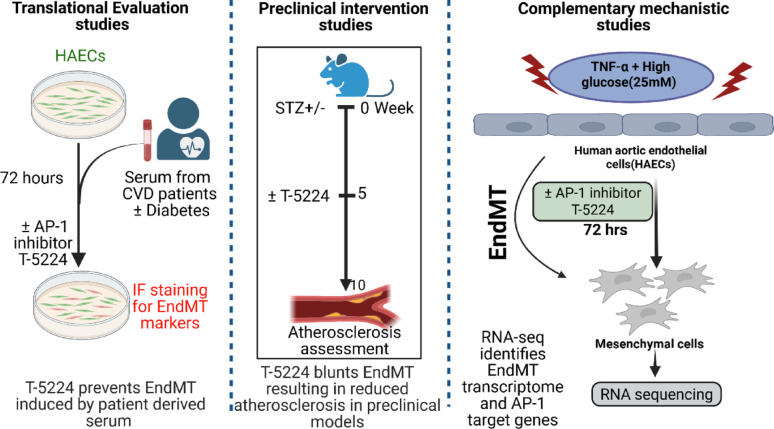

## Introduction

Endothelial to mesenchymal transition (EndMT) is a complex biological process where endothelial cells transform into a mesenchymal-like state. This transition plays a crucial role in various physiological and pathological conditions, including fibrosis, cancer, and cardiovascular disease [1]. EndMT is regulated by multiple factors, and transcription factors like activator protein 1 (AP-1) play a significant role. Recent studies have highlighted the involvement of EndMT in the development of atherosclerosis, a major contributor to cardiovascular disease-related deaths in individuals with diabetes [2]. However, the specific role of AP-1 in mediating EndMT in the context of atherosclerosis, particularly in diabetes, remains poorly understood.

Diabetes is a prevalent metabolic disorder that exacerbates the risk of atherosclerosis, leading to increased morbidity and mortality [2]. Current clinical interventions focus on reducing disease-promoting factors including hypercholesterolemia, hyperglycaemia, and hypertension. Despite these efforts, the residual atherosclerotic burden persists, as evident by the elevated atherosclerotic CVD-related mortality worldwide [3–5]. Thus, novel mechanism-based approaches are needed to directly inhibit the underlying pathobiology of atherosclerosis.

In our previous work, single cell RNA sequencing identified four vascular endothelial cell subpopulations [6]. One specific subpopulation was shown to originate from atheroprone aortic regions exposed to pro-atherogenic turbulent blood flow. This subpopulation exhibited a transcriptomic signature of EndMT, consistent with other single cell RNA sequencing studies [7–9]. Notably, AP-1 expression was exclusively increased by diabetes in this specific endothelial cell subpopulation, suggesting a connection between EndMT, diabetes and AP-1.^6^ Further microfluidic experiments in vascular endothelial cells demonstrated increased AP-1 activity under pro-atherogenic low shear stress and high glucose conditions [6]. Additionally, we showed that inhibiting AP-1 with the small molecule inhibitor T-5224 significantly attenuated atherosclerosis in a diabetic, atheroprone *Apoe*^−/−^ mouse model [6]. 

Building on these findings, this study explores the role of the AP-1 in EndMT associated with atherosclerosis in diabetes. We developed a unique translational approach to induce EndMT in primary vascular endothelial cells using serum isolated from patients with and without diabetes and tested whether AP-1 inhibition with T-5224 could prevent EndMT in this setting. Furthermore, the effects of T-5224 treatment on EndMT were assessed in the preclinical intervention study using a mouse model of diabetes-associated atherosclerosis. We also aimed to identify the transcriptomic signature of EndMT by conducting RNA sequencing in primary vascular endothelial cells under high glucose conditions and evaluated whether T-5224 treatment could prevent these gene expression changes, providing insights into vasculoprotective mechanisms afforded by T-5224. Through this comprehensive approach, we aim to uncover new therapeutic strategies to mitigate EndMT and its complications, including atherosclerosis in diabetes.

## Research design and methods

### Ethics statements

The preclinical experimental procedures were conducted in accordance with the ARRIVE guidelines and complied with the Australian National Health and Medical Research Council (NHMRC) standards. Study approval was obtained from the Alfred Medical Research Education Precinct (AMREP) Animal Services Ethics Committee (P8458 V1.3). For studies involving human serum, approval was granted by the Alfred Human Research Ethics Committee (authorisation number 232/17).

### Study population

All participants in this study (*n* = 19; 10 with T2DM and 9 without diabetes) were enrolled as part of the CARDINOX study. All had angiographically confirmed ASCVD. Detailed demographic and clinical characteristics are presented in Table 1. Pharmacological management included angiotensin II receptor blockers (ARBs), angiotensin-converting enzyme (ACE) inhibitors, acetylsalicylic acid (aspirin), and HMG-CoA reductase inhibitors (statins). Participants with concomitant T2DM received standard antidiabetic therapy in accordance with current clinical guidelines, including sodium-glucose co-transporter 2 (SGLT2) inhibitors, dipeptidyl peptidase-4 (DPP-4) inhibitors, and metformin.

**Table 1:** Demographic and clinical characteristics of study population

Parameters	Non-diabetic	Diabetic	*P* values
Male: Female (Total)	7:2 (9)	8:3 (10)	
Age	58.6 ± 9.4	67.4 ± 9.5	0.148
BMI	31.18 ± 7.7	31.67 ± 6.7	0.785
HbA1c (%)	5.52 ± 0.39	6.69 ± 1.04	0.002
Gensini score	16.66 ± 6.34	39.0 ± 20.8	0.006
Systolic BP (mmHg)	138 ± 16.7	151.8 ± 21.9	0.106
Diastolic BP (mmHg)	80.5 ± 21.9	77.4 ± 12.9	0.688
Total Cholesterol	3.40 ± 0.9	3.61 ± 0.7	0.528
HDL (mmol/l)	0.90 ± 0.17	1.01 ± 0.21	0.269
LDL (mmol/l)	1.93 ± 0.90	1.75 ± 0.49	0.795
Triglycerides (mmol/l)	1.21 ± 0.59	1.86 ± 1.11	0.372

Data are shown as mean ± SD. No significant difference was observed in age, BMI, blood pressure and plasma cholesterol or triglycerides levels between non-diabetic and diabetic patients with non-parametric two tailed Mann-Whitney test

### Experimental design for preclinical studies

Preclinical animal studies were conducted as previously described [6]. Briefly, male Apolipoprotein E deficient mice (*Apoe*^−/−^) on a C57Bl/6 background (Australia Resource Centre, Western Australia) were administered either streptozotocin (STZ, 55 mg/kg body weight) for diabetes induction or citrate vehicle for non-diabetic controls via intraperitoneal injections over 5 consecutive days. Body weights and blood glucose levels were monitored daily to confirm the diabetic status. After 5 weeks, the mice were randomised into four experimental groups: Control + Vehicle, Control + T-5224, Diabetes + Vehicle, Diabetes + T-5224 (*n* = 6–10 per group). The mice were then given either a vehicle (polyvinylpyrrolidone, PVP) or T-5224 dissolved in PVP (30 mg/kg body weight) daily by oral gavage for the next 5 weeks. T-5224 was administered orally at 30 mg/kg body weight/day, consistent with dosing used in prior murine studies showing effective AP-1 inhibition [10]. At the end of the study, animal tissues, including aortic roots were collected for analysis [11]. 

### Histology, immunohistochemistry and Immunofluorescence

At the conclusion of the preclinical study, aortic roots were harvested and embedded in optimal cutting temperature (OCT) compound. The frozen tissues were sectioned into 10 μm thick slices using a Leica cryostat and subsequently fixed with 4% paraformaldehyde for histological analysis. Atherosclerotic plaques in the aortic root were visualised with hematoxylin and eosin (H&E) staining as previously described [6]. Images were captured at 4x magnification using a microscope connected to a digital camera. Atherosclerotic lesions were quantified from digital microscope photograph using ImageJ software, calculating the relative lesion area to the total vessel area as previously described [11]. 

Quantitative data from three spatially separated sections were averaged to yield a single value per animal. For immunofluorescent staining, sections were incubated overnight at 4 °C with primary antibody (anti-CD31 ab256569, Abcam, 1:100; anti-FAP, ab218164, Abcam, 1:100; anti VE Cadherin antibody, ab33168, Abcam, 1:100; Anti-alpha smooth muscle Actin (α-SMA) antibody ab21027 Abcam, 1:100;). Following PBS washes, sections were stained with Cy5 and Texas Red conjugated secondary antibody (anti-rat or anti rabbit) for one hour at room Temperature. Nuclei were stained with Hoechst 33,342 nuclear staining dye (PureBlu™, 1351304EDU, Bio-Rad). Images were coded and then analysed for cell counting in a blinded manner. At least five images were evaluated from each of three spatially separated sinus sections per mouse. Data were averaged per animal and then used for statistical analyses.

### AP-1 activity assay

Nuclear protein was extracted using an acid extraction protocol as previously described [12]. Protein concentration was quantified, and equal amounts of nuclear protein were used to assess AP-1 DNA-binding activity using the AP1 Transcription Factor Assay Kit (ab207196, Abcam, Colorimetric) following manufacturer’s instructions.

### Cell culture, EndMT induction using human serum and under high glucose conditions, and shRNA-mediated FOS knockdown.

Primary Human aortic endothelial cells (HAECs; CC-2535) were obtained from LONZA (Mt Waverley, Australia) and cultured in EGM^TM^-2 media supplemented with Endothelial Cell Growth Medium-2 Bulletkit (CC-3156 and CC-4176, LONZA, Australia) at 37 °C in a 5% CO_2_ tissue culture incubator. For EndMT induction using patient serum, HAECs were cultured in EGM^TM^-2 media containing 10% serum from patients with and without diabetes in a 48-well plate for 72 h, in the presence and absence of the AP-1 inhibitor T-5224. Since all T2D patients were on diabetic medications with optimal glucose control, cells cultured in media containing diabetic serum were supplemented with glucose to a final concentration of 25 mM.

For EndMT induction under high glucose conditions, cells were cultured in a 12-well plate and stimulated with TNF-*α* (5 ng/mL) and high glucose (25 mM) for 72 h as previously described,^13^ with and without the T-5224. RNA-seq, *FOS* knockdown, and RT-PCR experiments were performed on cells from passages 3–8.

*FOS* knockdown was achieved by MISSION shRNA-expressing lentiviral vectors (Sigma-Aldrich) specifically targeting human *FOS* (RefSeq: NM_005252). The target sequence for *FOS* was 5’-GCGGAGACAGACCAACTAGAA-3’. Knockdown efficiency was verified by qRT-PCR with *FOS*-specific primers. Cells transduced with MISSION nontargeting shRNA vectors served as controls. These cells were subjected to the EndMT protocols described above.

### Immunofluorescent staining of cells

For immunofluorescent staining, cells were fixed with 4% paraformaldehyde for 10 min. The cells were then incubated overnight at 4 °C with primary antibody (anti-CD31, ab256569, Abcam, 1:100; anti-FAP, ab218164, Abcam, 1:100, anti VE Cadherin antibody ab33168, Abcam, 1:100; Anti- α-SMA antibody ab21027 Abcam, 1:100). Following washes with PBS, cells were stained with Cy5 and Texas Red conjugated secondary antibody (anti-rat, anti-goat or anti rabbit) for one hour at room Temperature. Nuclei were stained with Hoechst 33,342 nuclear staining dye (PureBlu™, 1351304EDU, Bio-Rad). Images were acquired with the EVOS cell imaging system (EVOS M5000, Thermo Fisher Scientific). Three to five random fields of view were imaged per well, and data were averaged to obtain a single value per replicate for statistical analyses. Digital micrographs were coded and analysed in a blinded manner using the colour threshold feature in ImageJ software.

### Quantification of FAP by ELISA

Soluble fibroblast activation protein (FAP) concentrations in human serum and cell culture supernatants were measured using a commercially available Human FAP ELISA Kit (Abcam, ab193701) according to the manufacturer’s instructions. Briefly, serum samples were diluted 1:200 in Assay Diluent A, and cell culture supernatants were diluted in 1× Assay Diluent B. Standards were prepared by serial dilution of recombinant FAP to generate a calibration curve ranging from 4,000 pg/mL to 16.4 pg/mL, with a blank included. A 96-well plate pre-coated with anti-human FAP antibody was loaded with 100 µL of standards or diluted samples and incubated for overnight at 4 °C with gentle agitation. After four washes with 1× Wash Buffer, 100 µL of biotinylated anti-FAP detection antibody was added and incubated for 1 h at room temperature. Following additional washes, 100 µL of HRP–streptavidin solution was applied for 45 min. Wells were washed again, and 100 µL of TMB substrate was added and incubated for 30 min in the dark. The reaction was terminated by adding 50 µL of Stop Solution, and absorbance was measured at 450 nm using a microplate reader. FAP concentrations were calculated by interpolation from the standard curve and corrected for sample dilution.

### Total RNA extraction

Total RNA was extracted from HAECs of all experimental groups using TRIzol reagent^®^ (Invitrogen Australia, Mt Waverley, Vic, Australia) and Direct-zol™ RNA miniprep kit (Zymo Research; Irvine CA, USA), following the manufacturer’s instructions as previously described [14]. The isolated RNA was then used for either RNA sequencing or RT PCR.

### RNA sequencing

Total RNA (1 µg) was subjected to ribosomal RNA depletion using NEBNext^®^ rRNA Depletion Kit module (New England Biolabs, Ipswich, MA). Barcoded libraries were generated using the NEBNext^®^ Ultra™ II Directional RNA Library Prep Kit for Illumina^®^ (New England Biolabs) according to the manufacturer’s instructions. Deep sequencing was performed using Illumina NovaSeq PE 75 (San Diego, CA) at the Novogen Company Limited, Hong Kong. Raw reads were trimmed for adaptor sequences and low quality bases using Skewer,^15^ and aligned to the human reference genome (GRCh38/hg38) using STAR aligner [16]. Tags aligning to genes were counted using Subread: FeatureCounts with Ensembl annotations (hg38-Ensembl Transcript release 103). Further analysis was conducted with Partek Flow software. Normalisation and differential gene expression (DGE) analyses were performed using the Gene Specific Analysis (GSA) method within Partek Flow. The false discovery rate (FDR) threshold of less than 0.05 and a fold change of at least ± 2 were considered statistically significant. Heatmaps and volcano plots for RNA-seq data were generated in Partek flow software. The Partek Flow’s pathways analysis tool was also utilized to assess the broader functional impact of these DEGs.

To visualise the relationship between gene expression and functional pathways, DGE and pathway enrichment results were obtained from Partek Genomics Suite and exported for visualization with R (v4.2.0) using ggplot2 and ggalluvial packages [17, 18]. A clustered bubble plot was constructed to display gene expression changes across experimental contrasts, where bubble size represented statistical significance (FDR) and colour indicated direction and fold change (FC). In parallel, pathway associations were visualized using an alluvial plot, linking genes to enriched pathways. The two plots were combined to depict gene-level differential expression and their functional pathway contexts.

### Quantitative PCR

Total RNA was reverse transcribed to cDNA using the high-capacity cDNA conversion kit (Applied Biosystems; Foster City, CA, USA). Transcript levels were quantified by RT-PCR using the FastStart Universal SYBR Green Master Mix (Roche; Melbourne Australia) on an ABI 7900HT PCR cycler (Applied Biosystems). Transcript levels of the genes of interest were normalized to the expression of the housekeeping gene *H3F3*. Relative quantification was calculated using the ΔCT formula. Primers used in RT-qPCR are listed in Supp Table S1.

### Statistical analyses

Statistical analyses were performed using GraphPad Prism Software (version 9.0.1). A two tailed student’s *t*-test for parametric data and the Mann Whitney test for non-parametric data were used to determine significance between two groups. One-way or Two-way Analysis of Variance (ANOVA) with Tukey’s multiple comparison and the Kruskal-Wallis with Dunn’s multiple comparison tests were used for comparisons among several groups of parametric and non-parametric data, respectively. A p-value of less than 0.05 was considered statistically significant. Data are presented as mean ± standard error of the mean (SEM).

### Data availability

All data supporting the findings of this study are available within the article and its supplemental material. The raw sequencing datasets can be provided upon reasonable request from the corresponding author.

## Results

### Serum from diabetic patients promotes EndMT, and AP-1 blockade by T-5224 prevents it

**Fig. 1:**
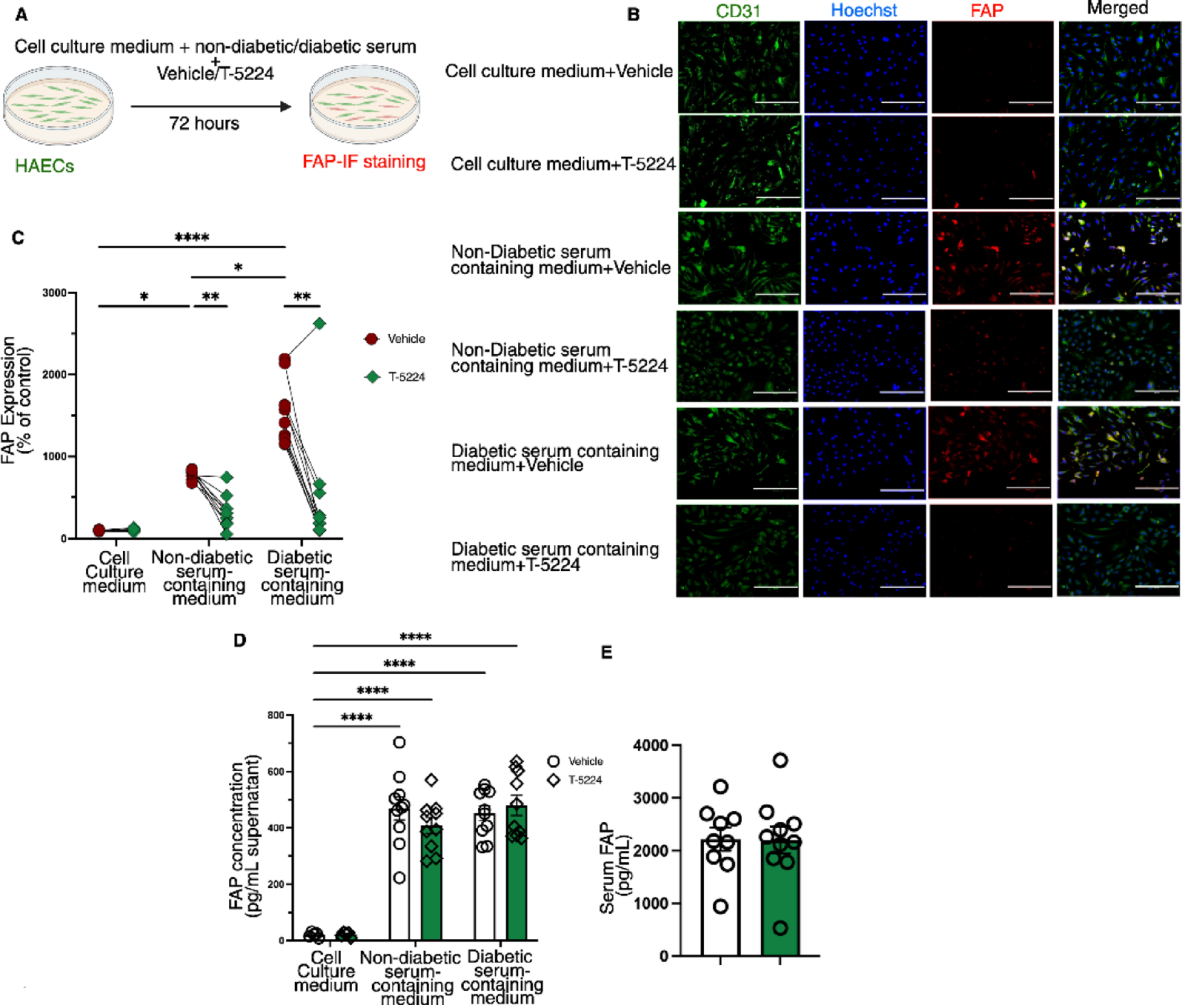
T-5224 alleviates EndMT induced by patient-derived serum. **A** Schematic illustration for ex vivo translational studies. **B** Dot plot with lines connecting individual biological replicates and **C** representative images of FAP immunofluorescent staining in human aortic endothelial cells (HAECs) cultured in media containing normal serum, patient-derived serum, with and without T-5224 (*n* = 9–10). The scale is 200 μm. **D** Soluble FAP levels measured by ELISA in culture supernatant (*n* = 9–10). **E** ELISA measurement of serum FAP levels in patients with and without diabetes (*n* = 9–10). Data are shown as mean ± SEM. *P* values were determined using a Two-way ANOVA with Tukey multiple comparison test. *=*P* < 0.05, **=*P* < 0.01, ***=*P* < 0.001, ****=*P* < 0.0001

We initially developed an *ex vivo* translational disease model where primary human aortic endothelial cells (HAECs) were exposed to patient-specific systemic/metabolic conditions using serum isolated from patients with and without diabetes. All patients recruited as part of the CARDINOX study had established atherosclerotic CVD as assessed by coronary angiogram. Study participants data are listed in Table 1. No significant difference was observed in age, BMI, blood pressure and plasma cholesterol or triglycerides levels between non-diabetic and diabetic individuals. The medications for these individuals included ARBs, ACE inhibitors, Aspirin, and statins. Additionally, individuals with diabetes were on standard therapy for diabetes including SGLT2 inhibitors, DPP-4 inhibitors, and metformin. The burden of atherosclerotic disease was calculated using the Gensini score which is a widely used angiographic scoring system for quantifying the severity and burden of coronary artery disease [19]. Diabetes was associated with significant increase in the Gensini score (Table 1). HAECs were cultured in serum containing media for 72 h in the presence and absence of AP-1 inhibitor, T-5224 (Fig. 1A). These cells were then evaluated for EndMT through immunofluorescent staining for the expression of fibroblast activation protein (FAP). FAP is a fibroblast-specific marker expressed by inflammatory- and cancer-associated stromal cells and fibroblasts, and it is also present in atherosclerotic plaques [20, 21]. Recent studies of genetically marked Fap^+ ve^ cells confirmed the absence of Fap expression in endothelial cells [22]. Fap has been previously used as an EndMT marker in both human and murine atherosclerosis [23]. Immunofluorescent analysis revealed a significant increase in FAP expression in HAECs exposed to serum of both diabetic and non-diabetic subjects, compared to control cell grown in normal cell culture media, indicating EndMT (Fig. 1B-C). Importantly, FAP expression was further increased significantly in HAECs exposed to serum of diabetic patients, compared to that of non-diabetic patients, indicating robust EndMT (Fig. 1B-C). Importantly, T-5224 treatment blocked EndMT, as evidenced by the significant reduction in FAP expression, in HAECs exposed to serum of both non-diabetic and diabetic patients, compared to those treated with the vehicle (Fig. 1B-C). We measured soluble FAP in cell culture media and observed significantly higher concentrations of FAP in media supplemented with either non-diabetic or diabetic serum as compared to standard culture media without serum (Fig. 1D). However, FAP concentration did not differ between cells treated with vehicle and those treated with T-5224 (Fig. 1D). Next, we assessed FAP levels in serum samples from non-diabetic and diabetic patients and observed no significant difference between the two groups (Fig. 1E). These findings suggest that the FAP detected in culture media originates from patients serum rather than being secreted by endothelial cells undergoing EndMT.

### EndMT occurs in the aortic roots of diabetic Apoe^−/−^ mice and T-5224 blunts EndMT

**Fig. 2:**
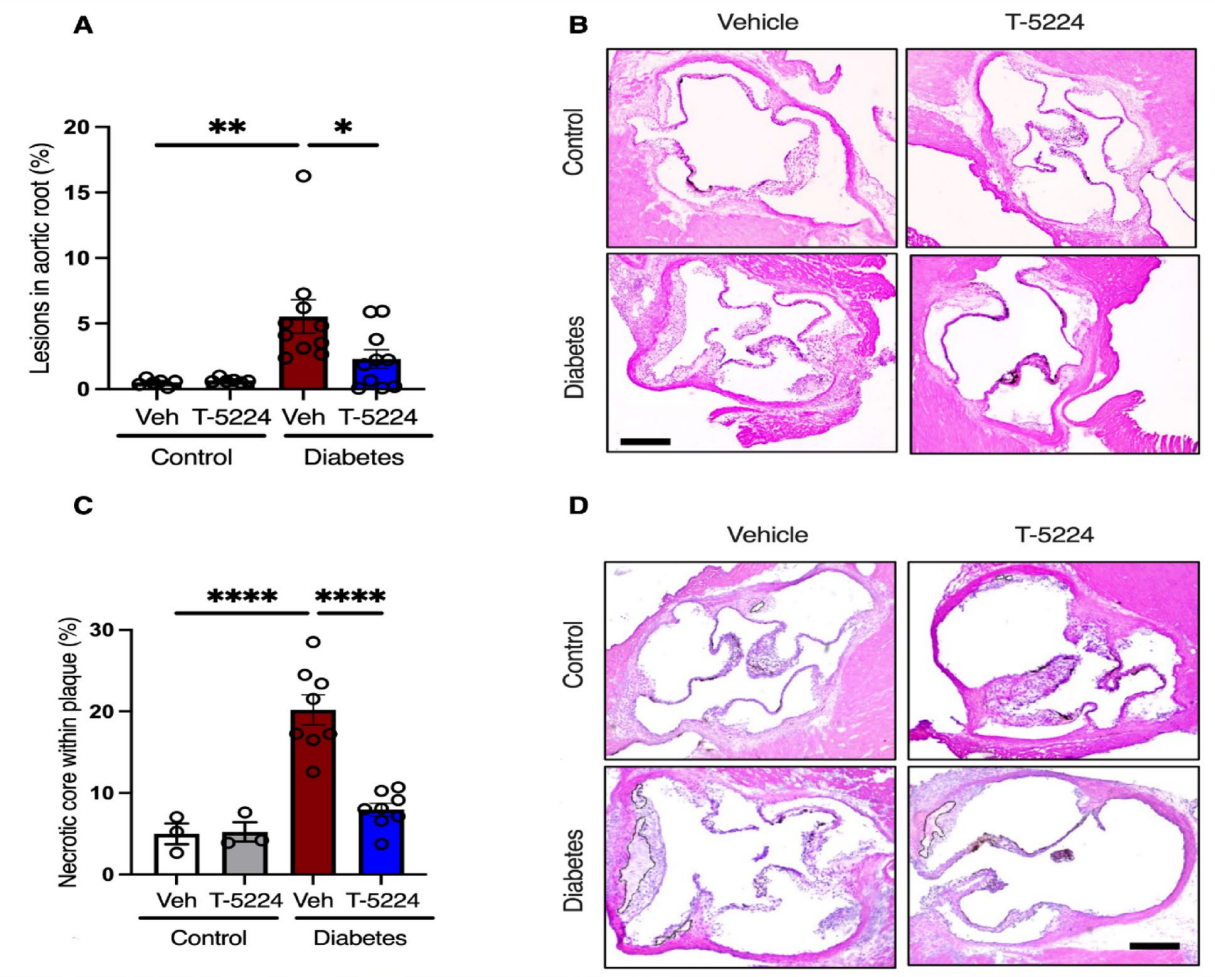
T-5224 treatment attenuates atherosclerosis in Diabetes in* Apoe*^−/−^ mice **A** Combined dot plot and bar graph for quantification of percent plaque area of aortic roots of control and diabetic *Apoe*^−/−^ mice treated either with vehicle or T-5224 (*n* = 6–10 per group). Data are shown as mean ± SEM. *P* value was determined using a one-way ANOVA with Tukey multiple comparison test. **B** Representative images of hematoxylin and eosin-stained aortic roots of control and diabetic *Apoe*^−/−^ mice treated either with vehicle or T-5224. Scale bars, 100 μm. **C** Combined dot plot and bar graph for quantification of percent necrotic core within plaques of aortic roots of control and diabetic *Apoe*^−/−^ mice treated either with vehicle or T-5224 (*n* = 3–8 per group). Data are shown as mean ± SEM. *P* value was determined using a one-way ANOVA with Tukey multiple comparison test. **D** Representative images of hematoxylin and eosin-stained aortic roots of control and diabetic *Apoe*^−/−^ mice treated either with vehicle or T-5224. Necrotic area is highlighted via encircled black lines. Scale bars, 100 μm. *=*P* < 0.05, **=*P* < 0.01, ***=*P* < 0.001, ****=*P* < 0.0001

**Fig. 3:**
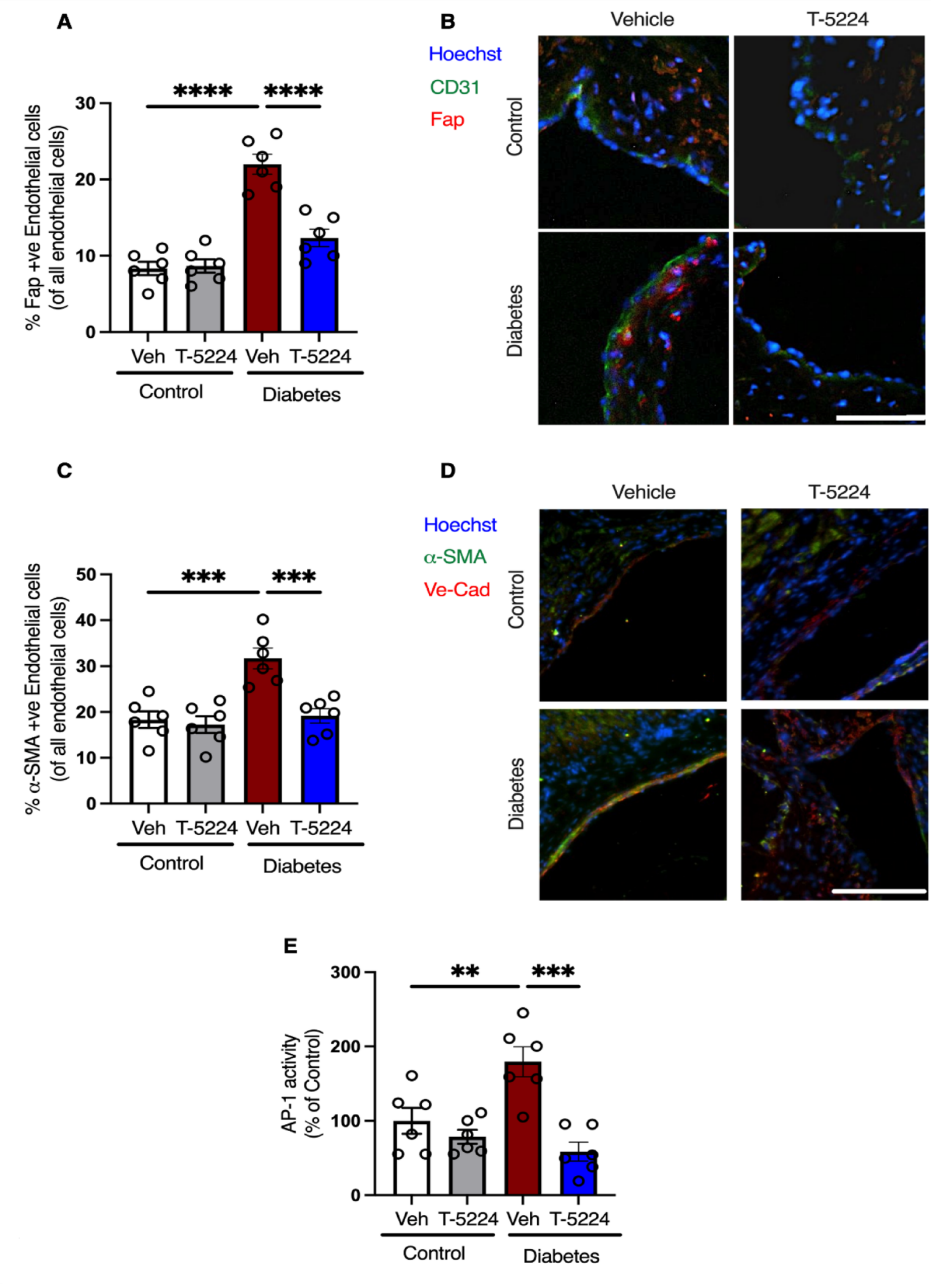
T-5224 treatment blocks EndMT in Diabetes, which triggers EndMT in the vascular endothelium of* Apoe*^−/−^ mice **A** Combined dot plot and bar graph for quantification of percent Fap^+ ve^ endothelial cells in aortic roots of control and diabetic *Apoe*^−/−^ mice treated either with vehicle or T-5224 (*n* = 6 per group). Data are shown as mean ± SEM. *P* values were determined using a one-way ANOVA with Tukey multiple comparison test. **B** Representative images of FAP immunofluorescent stained sections. The scale is 50 μm. **C** Combined dot plot and bar graph for quantification of percent α-SMA^+ ve^ endothelial cells in aortic roots of control and diabetic *Apoe*^−/−^ mice treated either with vehicle or T-5224 (*n* = 6 per group). Data are shown as mean ± SEM. *P* values were determined using a one-way ANOVA with Tukey multiple comparison test. **D** Representative images of α-SMA immunofluorescent stained sections. The scale is 100 μm. **E** Combined dot plot and bar graph for AP-1 activity (*n* = 6 per group). *=*P* < 0.05, **=*P* < 0.01, ***=*P* < 0.001, ****=*P* < 0.0001

We next evaluated the aortic roots for EndMT in a preclinical setting. Aortic roots were collected from a preclinical study in which diabetes was induced with streptozotocin (STZ) in atheroprone *Apoe*^−/−^ mice, and the mice were followed for 10 weeks. As anticipated, the aortic roots exhibited an increased atherosclerotic plaque area at 10 weeks of diabetes, consistent with the plaque observed in the aortic tree as previously reported in our recent study (Fig. 2A-B).^6^ Furthermore, AP-1 blockade by T-5224 significantly reduced atherosclerosis progression in diabetic *Apoe*^−/−^ mice, as evidenced by the lesional area in the aortic roots observed through H&E staining (Fig. 2A-B). Importantly, T-5224 treatment significantly reduced the necrotic area within diabetic plaques (Fig. 2C-D). We then evaluated EndMT in aortic sections through immunofluorescent staining for the co-expression of fibroblast activation protein (Fap). Immunofluorescence staining of plaques revealed a significant number of endothelial cells co-expressing Fap in the aortic roots of diabetic mice, compared to non-diabetic control mice, indicating EndMT (Fig. 3A-B). Importantly, T-5224 treatment blocked EndMT, as evidenced by the significant reduction in number of Fap-expressing endothelial cells in the aortic roots of diabetic mice when compared to vehicle-treated diabetic mice (Fig. 3A-B). To complement these findings, we assessed α-SMA expression in endothelial cells within the aortic sinus. Diabetic mice exhibited a marked increase in endothelial cells co-expressing α-SMA compared to non-diabetic control mice, further confirming EndMT (Fig. 3C-D). Notably, T-5224 treatment significantly reduced α-SMA-positive endothelial cells in diabetic mice relative to vehicle-treated diabetic mice (Fig. 3C-D), reinforcing its inhibitory effects on EndMT. To confirm effective AP-1 inhibition by T-5224, we assessed AP-1 DNA -binding activity in kidney tissue using a commercially available assay. As T-5224 was administered systemically, kidney tissue served as a surrogate for AP-1 activity, while aortic tissue was reserved for plaques analysis. Diabetic mice exhibited significantly higher AP-1 activity compared to non-diabetic controls, which was markedly reduced following T-5224 treatment (Fig. 3E).

### Gene expression profile of EndMT and AP-1 target genes under high glucose conditions

Given that AP-1 is a transcription factor complex regulating the transcription of hundreds of genes across multiple cellular pathways, we further explored the effects of AP-1 inhibition on the transcriptional signature of EndMT. This was achieved using an* in vitro* model of EndMT under high glucose conditions, shRNA-mediated gene knockdown strategies, and RNA sequencing.

### *In vitro* model of EndMT under high glucose conditions and pharmacological AP-1 inhibition using T-5224

**Fig. 4:**
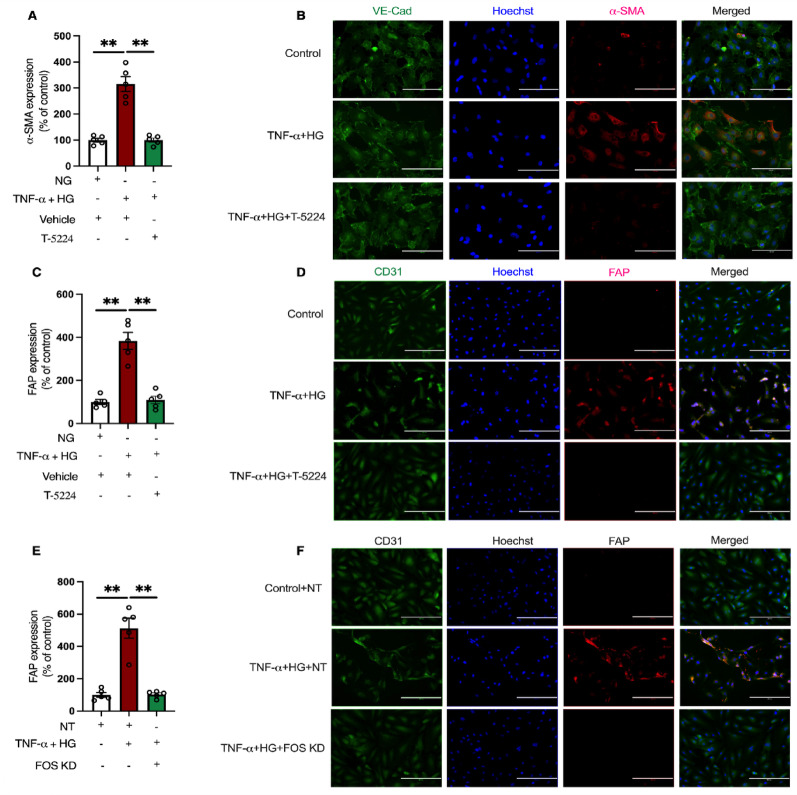
AP-1 inhibition with T-5224 blunts EndMT induction in the settings of TNF-α and high glucose in HAECs complemented with gene knockdown studies **A** Combined dot plot and bar graph and **B** representative images of α-SMA immunofluorescent staining in human aortic endothelial cells (HAECs) cultured in media containing TNF-α and high glucose, with and without T-5224 (*n* = 5). The scale is 100 μm. Data are shown as mean ± SEM. *P* values were determined using a one-way ANOVA with Tukey multiple comparison test. **C** Combined dot plot and bar graph and **D** representative images of FAP immunofluorescent staining in human aortic endothelial cells (HAECs) cultured in media containing TNF-α and high glucose, with and without T-5224 (*n* = 5). The scale is 200 μm. Data are shown as mean ± SEM. *P* values were determined using a one-way ANOVA with Tukey multiple comparison test. **E** Combined dot plot and bar graph and **F** representative images of FAP immunofluorescent staining in NT or *FOS* knockdown HAECs cultured in media containing TNF-α and high glucose (*n* = 5). The scale is 200 μm. Data are shown as mean ± SEM. *P* values were determined using a one-way ANOVA with Tukey multiple comparison test. *=*P* < 0.05, **=*P* < 0.01, ***=*P* < 0.001, ****=*P* < 0.0001

First, we tested whether AP-1 inhibition with T-5224 can attenuate EndMT* in vitro* using a well-established model [13]. This cell culture model involved primary vascular endothelial cells, HAECs, stimulated with TNF-α (5 ng/mL) under high glucose conditions, with and without T-5224. HAECs were cultured in media containing TNF-α and high glucose for 72 h in the presence and absence of T-5224. EndMT was then evaluated through immunofluorescent staining for α-SMA and FAP expression. Analysis revealed a marked increase in α-SMA expression in HAECs stimulated with TNF-α and high glucose compared to control cells grown under normal glucose conditions, indicating EndMT (Fig. 4A–B). Notably, T-5224 treatment significantly reduced α-SMA expression in HAECs exposed to TNF-α and high glucose compared to vehicle-treated cells (Fig. 4A–B). Consistent with these findings, FAP expression was also increased significantly by approximately 3-fold in HAECs stimulated with TNF-α and high glucose, compared to control cells grown under normal glucose conditions, confirming EndMT (Fig. 4C-D). Importantly, T-5224 stimulation blocked EndMT, as evidenced by the significant reduction in FAP expression in HAECs exposed to TNF-α and high glucose, compared to those treated with the vehicle (Fig. 4C-D).

### Complementary shRNA-mediated gene knockdown strategies

To further confirm these findings, complementary experiments involving shRNA-mediated gene knockdown strategies were performed. AP-1 is heterodimer complex composed of FOS, JUN and ATF, where FOS: JUN dimers have been reported to exhibit more robust DNA binding and transcriptional regulatory activity. Having demonstrated that AP-1 inhibition blocks EndMT, we investigated the effects of loss of one of the AP-1 members, FOS, on EndMT using lentiviral shRNA as a proof of principle. Lentiviral transduction of HAECs with FOS shRNA significantly reduced FOS expression compared to nontarget (NT) shRNA (Supp Fig. 1). HAECs with stable FOS knockdown and cells with NT shRNA were cultured in media containing TNF-α and high glucose for 72 h. Immunofluorescent staining for FAP expression was performed to evaluate EndMT. A significant increase in FAP expression was observed in NT HAECs stimulated with TNF-α and high glucose, compared to control NT cells grown under normal glucose conditions, reflecting EndMT (Fig. 4E-F). Importantly, EndMT was prevented in HAECs with stable FOS knockdown, as evidenced by the significant reduction in FAP expression in cells exposed to TNF-α and high glucose, compared to NT cells (Fig. 4E-F).

### RNA sequencing

**Fig. 5:**
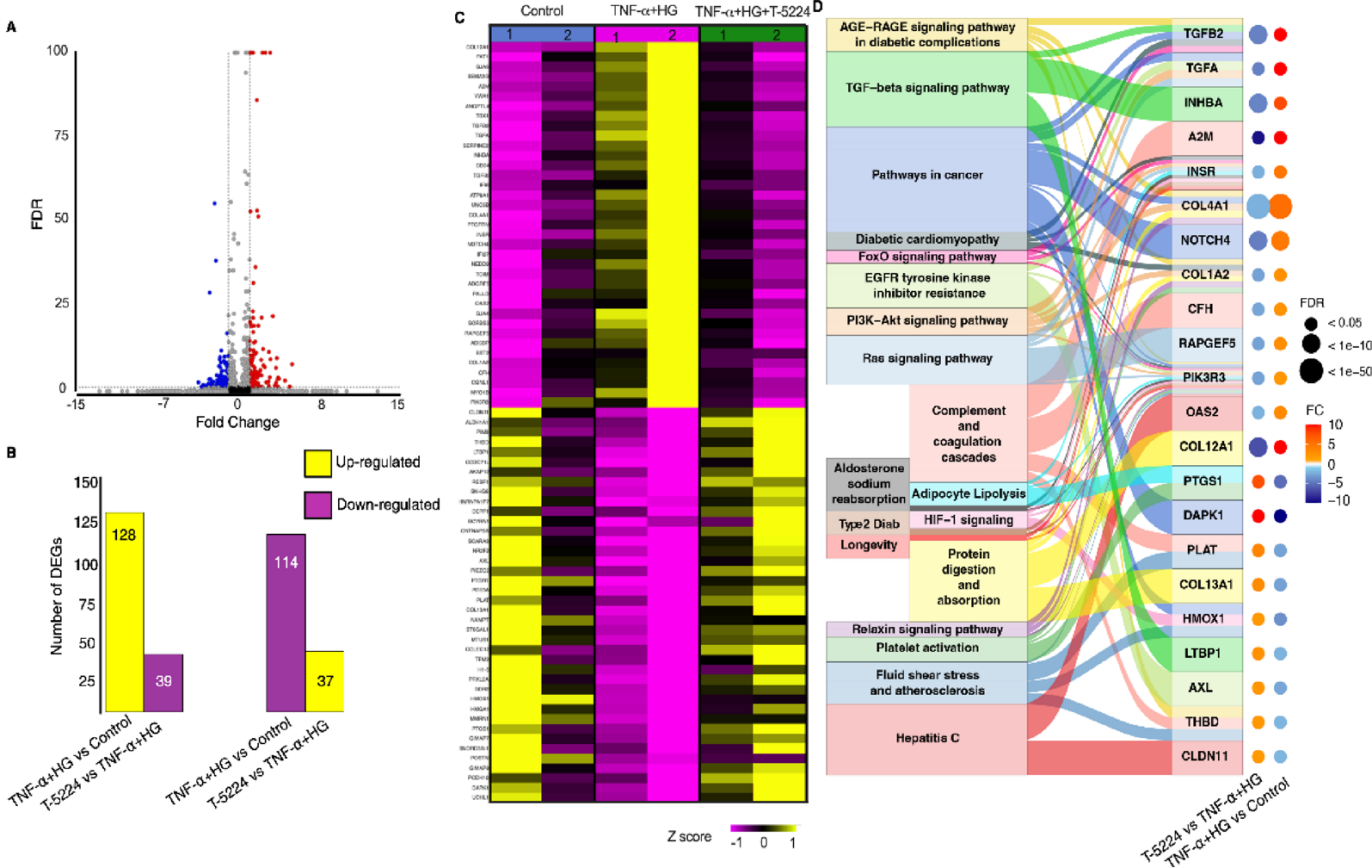
RNA-seq identified gene expression profile of EndMT and differentially expressed genes rescued by AP-1 inhibition with T-5224 in HAECs under high glucose conditions. **A** The volcano plot showing differential gene expression (DGE) profile associated with EndMT under high glucose conditions. A total of 242 genes were differentially expressed with at least a 2-fold change and a false discovery rate (FDR) of less than 0.05. **B** Among these, 128 were upregulated and 114 genes were downregulated compared to unstimulated cells. T-5224 treatment rescued the expression of 39 of 128 up-regulated genes and 37 of 114 down-regulated genes. **C** The heatmap of expression changes of differentially expressed genes in response to T-5224 treatment. T-5224 treatment rescued the expression of 77 genes despite the presence of TNF-α and high glucose. **D** Using the alluvial plot, the relationship of DEGs targeted by T-5224 treatment and their functional pathways in EndMT in shown. In addition, the bubble plot display gene expression changes across experimental conditions, where bubble size represented statistical significance (FDR) and colour indicates direction and fold change (FC)

After confirming that EndMT is blocked by AP-1 inhibition, either genetically using shRNA to target *FOS* or pharmacologically with T-5224, in HAECs, we aimed to identify gene expression changes associated with EndMT and to determine whether T-5224 treatment could reverse these changes. GSA identified a differential gene expression (DGE) profile associated with EndMT under high glucose conditions. Out of the 242 genes that were differentially expressed with at least a 2-fold change and FDR of less than 0.05, 114 were down-regulated and 128 were up-regulated compared to unstimulated cells (Fig. 5A-B and Supp Table S2). RNA-seq data was enriched for many genes previously reported to be linked to EndMT including *COL4A1*,* COL4A2*,* COL5A1*,* COL5A2*, *MMP2*,* FN1*,* TGF*α, *TGFβ1*,* TGFβ2* and *SERPINE2*. Expression of 77 of these differentially expressed genes (DEGs) (39 of 128 upregulated and 37 of 114 down-regulated genes) was rescued by T-5224 treatment despite the presence of TNF-α and high glucose (Fig. 5B-C and Supp Table S3).

**Fig. 6:**
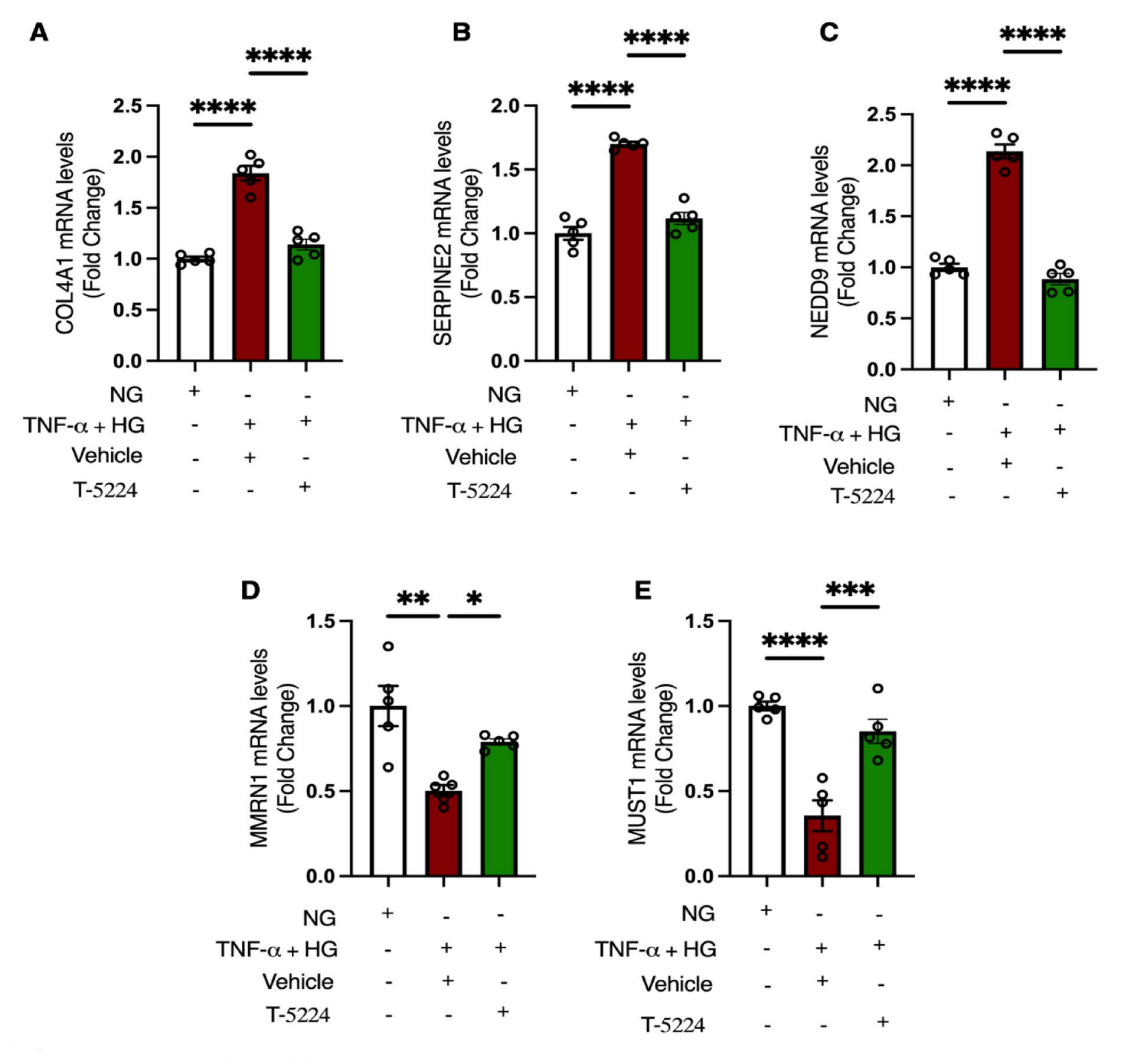
Validation of RNA-seq data by qPCR. The increase in expression of A) COL4A1, **B** SERPINE2, and **C** NEDD9 induced by TNF-α and high glucose was mitigated by T-5224 treatment (*n* = 5). The decrease in expression of **D** MMRN1 and **E** MUST1 caused by TNF-α and high glucose was alleviated by T-5224 treatment (*n* = 5). Data are shown as mean ± SEM. *P* value was determined using a one-way ANOVA with Tukey multiple comparison test. *=*P* < 0.05, **=*P* < 0.01, ***=*P* < 0.001, ****=*P* < 0.0001

T-5224 treatment reduced the expression of several EndMT-mediated upregulated genes, such as *COL4A1*, *TGFα*, *TGFβ2*, *NEDD9*, and *SERPINE2*, while increasing the expression of several EndMT-mediated downregulated genes, including *MMRN1*, *MUST1*, *CLDN11*, *ALDH1A1*, and *DEPP1* (Fig. 5C and Supp Table S3). We next performed KEGG pathway enrichment analysis with default settings in the Partek flow and identified several enriched pathways in HAECs where EndMT was blocked by T-5224 treatment. These pathways included the complement and coagulation cascade pathway, the AGE-RAGE signalling pathway in diabetic complications, the PI3K-Akt signalling pathway, the HIF-1 signalling pathway, and the fluid shear stress and atherosclerosis (Fig. 5D). The alluvial plot shows DEGs enriched in the top 22 pathways with several genes including *COL4A1 TGFα*, *TGFβ2* enriched in multiple pathways (Fig. 5D). The effects of AP-1 inhibition on expression of five of the EndMT-mediated differentially expressed genes as mentioned above were verified by qPCR. The increase in expression of *COL4A1*, *SERPINE2* and *NEDD9* induced by the TNF-α and high glucose was mitigated by T-5224 treatment, consistent with the RNA-seq data (Fig. 6A-C). Conversely, the decrease in expression of *MMRN*1 and *MUST1* caused by TNF-α and high glucose was abrogated by T-5224 treatment, in line with the RNA-seq data (Fig. 6D-E).

## Discussion

Our study investigated the role of AP-1 inhibition in preventing EndMT and its subsequent impact on atherosclerosis, using human ex vivo,* in vitro* and* in vivo* models. The results demonstrated that the AP-1 inhibitor T-5224 effectively mitigates EndMT induced by diabetic conditions, in association with reduced atherosclerotic progression. The use of patient-derived serum to induce EndMT in HAECs revealed that diabetic conditions per se significantly promote this transition, highlighting the pathological relevance of EndMT in diabetes-associated vascular complications in the human context. The ability of T-5224 to prevent EndMT, as evidenced by immunofluorescent staining, underscores the therapeutic potential of targeting AP-1 in mitigating these adverse effects. Our findings demonstrate that the inhibition of AP-1 with the small molecule inhibitor T-5224 effectively prevents EndMT, thereby reducing atherosclerosis in a diabetic mouse model. In our preclinical model, T-5224 treatment not only inhibited EndMT but led to a marked reduction in atherosclerotic plaque formation in *Apoe*^−/−^ mice. This finding is particularly significant as it suggests that AP-1 inhibition can directly impact the progression of atherosclerosis, offering a novel approach to managing this condition in diabetic patients.

Our observations on FAP complement recent findings that circulating FAP does not correlate with tissue expression,^24^ suggesting that soluble FAP detected in culture media largely originates from serum added to the media rather than endothelial secretion. This distinction is clinically relevant, as plasma FAP has been proposed as a prognostic marker but may not directly reflect endothelial activation. Beyond its role as a marker, FAP is increasingly recognized as a functional regulator of cardiovascular remodelling. Sun et al.^25^ showed that FAP inhibition promotes cardiac repair by stabilizing Brain natriuretic peptide (BNP) and enhancing angiogenesis, highlighting its therapeutic potential. While our study focuses on diabetic vasculature, these findings underscore the broader importance of FAP in fibrosis and vascular pathology.

RNA sequencing analysis provided deeper insights into the molecular mechanisms underlying AP-1-mediated EndMT. The identification of 242 DEGs associated with EndMT under high glucose conditions highlights the extensive transcriptional reprogramming involved. Importantly, the restoration of 77 DEGs by T-5224 treatment suggests that AP-1 inhibition can reverse key aspects of the EndMT transcriptional signature, further supporting the therapeutic potential of this drug. Pathways linked to these genes included the AGE-RAGE signalling pathway in diabetic complication, the fluid shear stress and atherosclerosis, and the complement and coagulation cascade, all with implications in atherosclerosis development and progression. RNA-seq data of EndMT was consistent with the previous studies, with numerous genes showing similar trend of expression as reported in those studies [7–9, 13]. 

The results of the ex vivo experiments using human serum derived from CVD patients suggest that conditions mimicking the* in vivo* environment facilitate EndMT, and that this process is exacerbated in the context of diabetes. This study is pioneering in its use of serum isolated from CVD patients to mimic the vascular environment, thereby facilitating the EndMT process. The evidence from these experiments indicates that EndMT is further exacerbated by the hyperactive serum from diabetic patients. Importantly, the inhibition of AP-1 is shown to be significant in the context of EndMT in CVD particularly in diabetes. This is the first study to utilize diabetic patient serum in this manner to investigate a pathological mechanism in a disease condition.

The findings align with previous research indicating that EndMT contributes significantly to atherosclerosis. Recently, Evrard et al. demonstrated that EndMT is prevalent in atherosclerotic lesions and is associated with plaque instability [23]. Similarly, Chen et al. highlighted the role of EndMT in atherosclerosis progression, emphasizing the involvement of inflammatory cytokines and oscillatory shear stress in activating TGF-β signalling, which promotes EndMT [26]. Single cell RNA sequencing has revealed endothelial populations potentially transitioning to mesenchymal cells in calcific aortic valve disease, accompanied by increased expression of multiple AP-1 family members [27]. Furthermore, a recent study demonstrated that AP-1 activation can directly drive EndMT in the context of tumour fibrotic stroma formation, mediated by RHBDF1 signalling [28]. Similarly, cell specific transcriptomic analysis of endothelial cells from diabetic mouse heart and aorta identified distinct endothelial cell populations expressing mesenchymal markers, supporting the concept of endothelial plasticity during atherosclerosis development in diabetes [9]. These findings collectively suggest that AP-1 may serve as a critical transcriptional regulator of EndMT under pathological conditions, linking inflammatory and stress signalling to vascular remodelling. Our study extends these findings by showing that diabetes further exacerbated EndMT and that AP-1 inhibition can counteract these processes, suggesting a potential therapeutic avenue. The RNA sequencing data indicates that AP-1 plays a crucial role in regulating the transcriptional landscape of EndMT. The restoration of gene expression by T-5224 suggests that AP-1 inhibition can reverse the molecular changes induced by high glucose and the associated inflammatory milieu, thereby preventing EndMT and its pathological sequelae. These findings are particularly significant from clinical perspective given the challenges in managing atherosclerosis in diabetic patients, who are at higher risk for cardiovascular events. By targeting AP-1, it may be possible to develop new treatments that specifically address the underlying mechanisms driving atherosclerosis in this population.

The use of patient-derived serum in the current study provides a realistic model that closely mimics the* in vivo* environment, thereby enhancing the applicability of the results to human disease. This approach allows us to better understand the pathological mechanisms at play in diabetes-associated vascular complications. Our findings identify AP-1 as a pivotal regulator of EndMT in diabetes-associated atherosclerosis and demonstrate that its inhibition with T-5224 offers a promising strategy for reducing vascular complications in diabetic patients. Future research should focus on elucidating the precise molecular pathways through which AP-1 regulates EndMT and exploring the clinical applicability of AP-1 inhibitors in managing atherosclerosis in diabetes. While our study provides compelling evidence for the role of AP-1 in EndMT and atherosclerosis, there are limitations that need to be addressed in future research. The* in vitro* models, while informative, do not fully replicate the complexity of the* in vivo* environment. Additionally, the investigation of EndMT is based on immunofluorescent staining of an EndMT marker, and thus further lineage tracking studies are required to validate and confirm the origin of atherosclerosis associated fibroblast-like cells. Although atheroprotective effects of T-5224 were reproduced by the shRNA-based FOS knockdown studies, targeting other AP-1 members is warranted in additional studies. While plasma concentrations of T-5224 were not directly measured in this study, the selected dose (30 mg/kg/day) was based on prior evidence showing that oral administration of 30 mg/kg achieves effective AP-1 inhibition and therapeutic efficacy in mice [10]. Nevertheless, we recognize that without direct measurements, variability in systemic exposure cannot be excluded. Future studies should incorporate pharmacokinetic profiling to confirm plasma concentrations and correlate them with biological outcomes. Although our serum-based ex vivo studies provide proof-of-principle evidence that diabetic vascular conditions exacerbate EndMT and that AP-1 inhibition shows vasculoprotective effects, additional studies are required to thoroughly evaluate the long-term effects and safety of AP-1 inhibition in clinical settings. Our CARDINOX study population included only patients with established atherosclerotic CVD. Although our primary focus was on diabetes-associated EndMT, it is plausible that factors present in non-diabetic serum of patients with CVD could also contribute to increased FAP expression. Such factors may include inflammatory cytokines including TNF-α and IL-6, oxidative stress mediators, lipid-derived molecules, and metabolic stress markers, which can be elevated even in patients without diabetes due to other cardiovascular risk factors. In addition, we cannot exclude potential drug-mediated effects on EndMT. ARBs, DPP4 inhibitors, and SGLT2 inhibitors are routinely prescribed for patients with diabetes and cardiovascular disease, making it practically impossible to obtain drug-naïve samples in this population. While our experimental design ensured uniform processing of all sera and demonstrated that AP-1 inhibition with T-5224 consistently reduced EndMT markers in HAECs exposed to both diabetic and non-diabetic serum, we cannot completely exclude drug-mediated effects. Therefore, our findings should be interpreted in the context of this clinical reality. Future studies should also explore the potential synergistic effects of combining AP-1 inhibitors with other therapeutic agents to enhance efficacy.

In conclusion, our study demonstrates that AP-1 inhibition via T-5224 effectively prevents EndMT and reduces atherosclerosis in diabetic conditions. These findings provide a strong rationale for further investigation into AP-1 inhibitors as a therapeutic strategy for managing atherosclerosis, particularly in patients with diabetes. By targeting the molecular pathways involved in EndMT, it may be possible to develop more effective treatments that address the root causes of atherosclerosis and thereby improve cardiovascular outcomes.

## Supplementary Information

Below is the link to the electronic supplementary material.

Supplementary Material 1

## Data Availability

All data supporting the findings of this study are available within the article and its supplemental material. The raw sequencing datasets can be provided upon reasonable request from the corresponding author.
