# Supplementary material for: Activator protein-1 (AP-1) inhibition prevents endothelial to mesenchymal transition in diabetes-associated atherosclerosis: a translational study
Source: Cardiovasc Diabetol. 2026 Jan 29;25:61. doi: 10.1186/s12933-025-03060-5 (PMC12922352; doi:10.1186/s12933-025-03060-5)
Supplement: Supplementary file 1 — Supplementary Material 1 [file 12933_2025_3060_MOESM1_ESM.pdf]

Supplemental Material

Supp Fig 1.

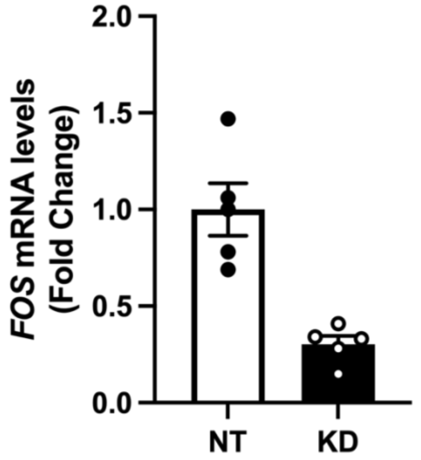

Supp Fig 1. FOS mRNA levels in non-target shRNA and FOS-specific shRNA induced FOS knock down in HAECs.

Supp Table S1

**Gene name**	**Forward sequence**	**Reverse sequence**
COL4A1	AAAGGGAGATCAAGGGAATAG	TCACCTTTTTCTCCAGGTAG
SERPINE2	CTACAGGTGCTGTGTTATTC	CTTTCTTCGTAGCAAAGTAGTC
NEDD9	ACAAGAGGTATATCAGGTGC	TTATCACCTTTTTACCCACG
MMRN1	ACGTGCATAAATGGAAGAAC	TCTGGAGCTAAAGCATTTTC
MUST1	AATGCAGTTGAAAAGAGCAG	CTGCAGGATAAATCCACTTTC
H3F3	GGTGTCTTCAAAAAGGCCAA	GCGAGAAATTGCTCAGGACT

Supp Table S2

Gene ID	Gene name	FDR step up (TNF-α + HG vs Control)	Fold change (TNF-α + HG vs Control)
COL4A1	COL4A1	0.00E+00	5.56E+00
COL4A2	COL4A2	6.98E-307	4.56E+00
FN1	FN1	4.36E-253	2.23E+00
SPARC	SPARC	3.38E-126	2.10E+00
APLN	APLN	5.57E-120	2.51E+00
CD34	CD34	7.51E-101	7.62E+00
MMP2	MMP2	1.03E-86	3.21E+00
MMRN1	MMRN1	3.35E-56	-4.86E+00
AGRN	AGRN	4.42E-54	3.26E+00
IGFBP7	IGFBP7	7.71E-54	2.11E+00
PXDN	PXDN	2.39E-52	3.51E+00
HMGA1	HMGA1	2.43E-39	-4.48E+00
COL5A2	COL5A2	2.24E-37	2.93E+00
COL5A1	COL5A1	1.00E-32	2.54E+00
POSTN	POSTN	7.59E-30	-6.68E+00
DKK3	DKK3	2.42E-24	2.48E+00
SERPINE2	SERPINE2	6.19E-23	9.04E+00
IFI27	IFI27	1.81E-22	4.35E+00
HLA-B	HLA-B	2.07E-22	2.52E+00
FSTL1	FSTL1	3.22E-21	2.03E+00
TUBA1A	TUBA1A	1.04E-20	2.11E+00
JAG2	JAG2	3.79E-20	3.53E+00
AD000090.1	AD000090.1	3.99E-20	2.68E+00
QSOX1	QSOX1	6.06E-20	2.25E+00
HTRA1	HTRA1	3.33E-18	2.52E+00
AKAP12	AKAP12	7.34E-18	-2.17E+00
RRBP1	RRBP1	1.37E-15	2.00E+00
ABI3BP	ABI3BP	2.08E-15	2.88E+00
RNU5A-1	RNU5A-1	4.05E-15	2.63E+00
NOTCH4	NOTCH4	3.21E-13	4.78E+00
SYNPO	SYNPO	5.95E-13	2.05E+00
CGNL1	CGNL1	7.06E-13	2.57E+00
MTUS1	MTUS1	8.75E-13	-3.14E+00
DCHS1	DCHS1	1.11E-12	2.58E+00
IFI6	IFI6	2.39E-12	7.08E+00
JAG1	JAG1	4.32E-12	3.09E+00
PTPRM	PTPRM	8.65E-12	2.90E+00
IL32	IL32	1.01E-11	2.64E+00
RNASE1	RNASE1	2.32E-11	2.16E+00
TKT	TKT	3.59E-11	-2.03E+00
NR2F2	NR2F2	6.24E-11	-2.56E+00
TNFRSF21	TNFRSF21	8.49E-11	2.49E+00
H1-5	H1-5	8.49E-11	-3.44E+00
ANGPTL4	ANGPTL4	1.65E-10	1.17E+01

TMEM132A	TMEM132A	2.61E-10	2.44E+00
INHBA	INHBA	3.28E-10	7.75E+00
PRXL2A	PRXL2A	4.02E-10	-3.51E+00
NEDD9	NEDD9	4.77E-10	4.24E+00
TMSB4XP6	TMSB4XP6	5.82E-10	2.12E+00
SOX17	SOX17	9.65E-10	2.35E+00
AXL	AXL	1.24E-09	-2.66E+00
RNU5B-1	RNU5B-1	1.57E-09	2.23E+00
PCDH1	PCDH1	1.64E-09	2.07E+00
SLC7A11	SLC7A11	2.35E-09	-2.47E+00
MKI67	MKI67	3.54E-09	-3.24E+00
MT-ATP8	MT-ATP8	4.07E-09	-2.10E+00
ANP32B	ANP32B	5.14E-09	-2.47E+00
CCL2	CCL2	6.81E-09	2.25E+00
COL12A1	COL12A1	1.02E-08	3.13E+01
TFPI	TFPI	1.07E-08	-2.14E+00
MDK	MDK	1.85E-08	2.22E+00
INSR	INSR	2.39E-08	4.89E+00
HMOX1	HMOX1	3.66E-08	-3.67E+00
HNRNPA1P7	HNRNPA1P7	3.88E-08	-2.19E+00
CFH	CFH	4.73E-08	2.61E+00
PTGFRN	PTGFRN	7.18E-08	5.35E+00
H3C2	H3C2	8.50E-08	-3.38E+00
BST2	BST2	9.72E-08	2.74E+00
TGFB2	TGFB2	1.15E-07	1.10E+01
CLDN11	CLDN11	3.48E-07	-2.01E+00
TCIM	TCIM	5.67E-07	4.04E+00
DEPP1	DEPP1	6.11E-07	-2.24E+00
COL1A2	COL1A2	6.56E-07	2.71E+00
NT5E	NT5E	7.17E-07	2.05E+00
H4C14	H4C14	8.72E-07	-2.91E+00
H3C3	H3C3	1.36E-06	-3.07E+00
HNRNPA1	HNRNPA1	1.78E-06	-2.18E+00
SORBS2	SORBS2	1.93E-06	3.20E+00
EFNB2	EFNB2	1.95E-06	3.16E+00
THSD7A	THSD7A	2.58E-06	2.64E+00
NQO1	NQO1	3.38E-06	-2.07E+00
CDH2	CDH2	3.64E-06	2.70E+00
ICAM1	ICAM1	3.89E-06	2.29E+00
SLC16A3	SLC16A3	3.91E-06	2.13E+00
RGCC	RGCC	3.98E-06	3.64E+00
H1-2	H1-2	5.90E-06	-2.02E+00
MRC2	MRC2	6.89E-06	2.33E+00
ISG15	ISG15	1.06E-05	2.94E+00
HELZ2	HELZ2	1.48E-05	2.37E+00
TPM2	TPM2	1.58E-05	-3.32E+00
H2BC18	H2BC18	1.90E-05	-4.23E+00
HMGB2	HMGB2	1.94E-05	-3.25E+00

SLC2A1	SLC2A1	2.00E-05	4.34E+00
ADGRF5	ADGRF5	2.02E-05	3.58E+00
GAS6	GAS6	2.12E-05	2.08E+00
ST6GAL1	ST6GAL1	2.49E-05	-3.12E+00
PCDH12	PCDH12	3.26E-05	3.22E+00
ADGRA2	ADGRA2	3.55E-05	2.38E+00
RAPGEF5	RAPGEF5	3.58E-05	2.98E+00
NAMPT	NAMPT	3.93E-05	-2.96E+00
PIEZO2	PIEZO2	4.26E-05	-2.67E+00
PTGR1	PTGR1	4.30E-05	-2.67E+00
H2AC19	H2AC19	6.21E-05	-2.48E+00
CD276	CD276	7.20E-05	2.18E+00
TGFBI	TGFBI	7.34E-05	7.42E+00
DHCR24	DHCR24	8.50E-05	-2.23E+00
H2BC11	H2BC11	8.56E-05	-3.60E+00
OAS2	OAS2	9.41E-05	3.31E+00
A2M	A2M	9.64E-05	1.27E+01
H2BC12	H2BC12	1.14E-04	-2.20E+00
CENPF	CENPF	1.14E-04	-3.15E+00
CYTL1	CYTL1	1.29E-04	2.68E+00
H4C2	H4C2	1.33E-04	-2.61E+00
RGS4	RGS4	1.36E-04	-2.91E+00
H2AC11	H2AC11	1.68E-04	-3.43E+00
SEMA3G	SEMA3G	1.93E-04	1.35E+01
H3C4	H3C4	2.02E-04	-3.36E+00
METTL7A	METTL7A	2.51E-04	-6.34E+00
VWA1	VWA1	2.72E-04	1.23E+01
GIMAP8	GIMAP8	2.74E-04	-6.90E+00
PIM3	PIM3	3.07E-04	-2.08E+00
ABHD17A	ABHD17A	3.76E-04	2.09E+00
SMPD1	SMPD1	4.43E-04	2.28E+00
BCYRN1	BCYRN1	4.49E-04	-2.30E+00
ALPK3	ALPK3	4.61E-04	2.07E+00
SNHG5	SNHG5	4.62E-04	-2.19E+00
MT1E	MT1E	5.22E-04	-4.10E+00
OGFRL1	OGFRL1	5.22E-04	2.51E+00
IPO5	IPO5	5.89E-04	-2.13E+00
SNCA	SNCA	6.18E-04	-2.44E+00
RESF1	RESF1	6.41E-04	-2.18E+00
VTRNA1-1	VTRNA1-1	6.50E-04	2.45E+00
GJA5	GJA5	6.69E-04	1.99E+01
TMPO	TMPO	7.76E-04	-2.36E+00
AKR1B1	AKR1B1	8.90E-04	-2.27E+00
PRR11	PRR11	1.13E-03	-3.41E+00
UNC5B	UNC5B	1.18E-03	6.81E+00
THBD	THBD	1.44E-03	-2.08E+00
PTGS1	PTGS1	1.44E-03	-5.29E+00
P4HA1	P4HA1	1.45E-03	2.90E+00

MYO1B	MYO1B	1.46E-03	2.51E+00
ADAMTSL1	ADAMTSL1	1.46E-03	2.50E+00
SRPX	SRPX	1.51E-03	-2.21E+00
H4C8	H4C8	1.63E-03	-2.68E+00
DDR2	DDR2	1.73E-03	-3.57E+00
TOP2A	TOP2A	1.85E-03	-3.04E+00
ALDH1A1	ALDH1A1	1.88E-03	-2.04E+00
MX1	MX1	2.13E-03	5.85E+00
UCHL1	UCHL1	2.16E-03	-1.39E+01
GIMAP7	GIMAP7	2.25E-03	-5.81E+00
COL13A1	COL13A1	2.36E-03	-2.87E+00
TPX2	TPX2	2.65E-03	-3.16E+00
SCARA3	SCARA3	2.95E-03	-2.55E+00
TBX1	TBX1	3.36E-03	1.10E+01
PLAT	PLAT	3.57E-03	-2.86E+00
ASPM	ASPM	3.73E-03	-4.13E+00
RNF152	RNF152	3.78E-03	2.20E+00
MN1	MN1	3.90E-03	3.46E+00
H2AC16	H2AC16	4.12E-03	-3.54E+00
TUBB3	TUBB3	4.65E-03	2.55E+00
H3C12	H3C12	4.86E-03	-7.19E+00
PRKAR2B	PRKAR2B	4.92E-03	-3.08E+00
EPB41L3	EPB41L3	5.03E-03	2.09E+00
SHMT2	SHMT2	5.11E-03	-2.35E+00
ADGRL2	ADGRL2	5.55E-03	2.10E+00
H2AC14	H2AC14	5.59E-03	-3.35E+00
HNRNPA1P10	HNRNPA1P10	5.83E-03	-2.11E+00
CCDC71L	CCDC71L	6.34E-03	-2.16E+00
CNTNAP3B	CNTNAP3B	6.34E-03	-2.39E+00
HNRNPA1L2	HNRNPA1L2	6.59E-03	-2.53E+00
SULF1	SULF1	6.83E-03	3.18E+00
LMNB1	LMNB1	6.88E-03	-2.62E+00
TENM3	TENM3	7.04E-03	2.27E+00
H2AC4	H2AC4	7.12E-03	-4.05E+00
CNKSR3	CNKSR3	8.39E-03	2.66E+00
PRC1	PRC1	8.50E-03	-3.04E+00
CHST1	CHST1	8.62E-03	2.48E+00
H2BC5	H2BC5	8.76E-03	-2.11E+00
RASGRF2	RASGRF2	8.76E-03	3.29E+00
EFEMP2	EFEMP2	8.80E-03	2.16E+00
SNORD3B-1	SNORD3B-1	9.49E-03	-6.26E+00
RNU12	RNU12	9.72E-03	2.02E+00
NAMPTP1	NAMPTP1	1.00E-02	-3.02E+00
H2AC7	H2AC7	1.06E-02	-2.42E+00
PSAT1	PSAT1	1.10E-02	-6.18E+00
H3C15	H3C15	1.12E-02	-2.56E+00
H3C14	H3C14	1.12E-02	-2.56E+00
P4HA2	P4HA2	1.25E-02	2.49E+00

CCDC85C	CCDC85C	1.28E-02	2.26E+00
ANLN	ANLN	1.33E-02	-2.79E+00
DAPK1	DAPK1	1.36E-02	-1.03E+01
H3C13	H3C13	1.37E-02	-4.32E+00
H3C11	H3C11	1.37E-02	-2.39E+00
CRACDL	CRACDL	1.40E-02	2.92E+00
CARD16	CARD16	1.47E-02	2.08E+00
PHGDH	PHGDH	1.48E-02	-9.63E+00
BMP1	BMP1	1.52E-02	2.24E+00
SOX13	SOX13	1.55E-02	2.32E+00
PALLD	PALLD	1.55E-02	3.49E+00
H3C10	H3C10	1.63E-02	-2.93E+00
H2AC12	H2AC12	1.67E-02	-3.17E+00
SNORA63	SNORA63	1.70E-02	2.06E+00
GJA4	GJA4	1.83E-02	3.30E+00
H2BC14	H2BC14	1.83E-02	-5.48E+00
ATP8A1	ATP8A1	1.90E-02	7.00E+00
TGFA	TGFA	1.96E-02	1.01E+01
PCDH10	PCDH10	2.01E-02	-8.33E+00
FAM89A	FAM89A	2.05E-02	3.58E+00
PMEPA1	PMEPA1	2.18E-02	2.55E+00
MGST1	MGST1	2.29E-02	-3.00E+00
LAMC2	LAMC2	2.37E-02	2.38E+00
TSPYL2	TSPYL2	2.53E-02	-3.02E+00
MTHFD2	MTHFD2	2.61E-02	-2.72E+00
CDC20	CDC20	2.65E-02	-4.68E+00
COLEC12	COLEC12	2.70E-02	-3.21E+00
H2AC17	H2AC17	2.70E-02	-2.34E+00
CDCA7L	CDCA7L	2.70E-02	-2.56E+00
HTR1B	HTR1B	2.76E-02	-5.25E+00
F2RL1	F2RL1	2.99E-02	2.49E+00
TUBB2A	TUBB2A	3.43E-02	2.36E+00
PDE3A	PDE3A	3.50E-02	-2.77E+00
H4C4	H4C4	3.64E-02	-2.20E+00
SMC4	SMC4	3.65E-02	-2.19E+00
EPB41L1	EPB41L1	3.71E-02	2.82E+00
CCNB1	CCNB1	3.76E-02	-3.21E+00
RIPK2	RIPK2	3.88E-02	2.53E+00
FAT1	FAT1	3.88E-02	2.69E+01
A4GALT	A4GALT	3.88E-02	2.26E+00
MMP15	MMP15	3.89E-02	2.95E+00
PIK3R3	PIK3R3	4.06E-02	2.23E+00
H2BC21	H2BC21	4.08E-02	-2.14E+00
AC073140.1	AC073140.1	4.14E-02	3.03E+00
NOG	NOG	4.14E-02	1.25E+01
FKBP5	FKBP5	4.14E-02	-2.02E+00
LTBP1	LTBP1	4.21E-02	-2.14E+00
HES4	HES4	4.22E-02	4.44E+00

MT-TV	MT-TV	4.24E-02	3.92E+00
ARHGAP11A	ARHGAP11A	4.35E-02	-2.87E+00
ZBTB16	ZBTB16	4.48E-02	-1.15E+01
NCAPD2	NCAPD2	4.76E-02	-2.23E+00
H2BC17	H2BC17	4.77E-02	-2.80E+00
SELENOM	SELENOM	4.78E-02	2.21E+00

Supp Table S3

Gene ID	Gene name	FDR step up (TNF-α + HG vs Control)	Fold change (TNF-α + HG vs Control)	FDR step up (T-5224 vs TNF-α + HG)	Fold change (T-5224 vs TNF-α + HG)
COL4A1	COL4A1	0.00E+00	5.56E+00	4.42E-221	-2.26E+00
CD34	CD34	7.51E-101	7.62E+00	6.41E-95	-4.24E+00
MMRN1	MMRN1	3.35E-56	-4.86E+00	3.43E-29	3.28E+00
HMGA1	HMGA1	2.43E-39	-4.48E+00	1.61E-25	3.47E+00
POSTN	POSTN	7.59E-30	-6.68E+00	2.27E-04	2.28E+00
SERPINE2	SERPINE2	6.19E-23	9.04E+00	3.70E-24	-4.29E+00
IFI27	IFI27	1.81E-22	4.35E+00	6.51E-24	-2.91E+00
AKAP12	AKAP12	7.34E-18	-2.17E+00	4.33E-60	3.72E+00
ABI3BP	ABI3BP	2.08E-15	2.88E+00	1.37E-18	-3.41E+00
NOTCH4	NOTCH4	3.21E-13	4.78E+00	7.78E-12	-4.47E+00
CGNL1	CGNL1	7.06E-13	2.57E+00	1.50E-09	-2.15E+00
MTUS1	MTUS1	8.75E-13	-3.14E+00	2.30E-10	2.85E+00
IFI6	IFI6	2.39E-12	7.08E+00	1.46E-15	-3.95E+00
NR2F2	NR2F2	6.24E-11	-2.56E+00	6.55E-11	2.56E+00
H1-5	H1-5	8.49E-11	-3.44E+00	1.57E-03	2.13E+00
ANGPTL4	ANGPTL4	1.65E-10	1.17E+01	1.06E-07	-2.66E+00
INHBA	INHBA	3.28E-10	7.75E+00	2.12E-13	-4.21E+00
PRXL2A	PRXL2A	4.02E-10	-3.51E+00	7.50E-12	3.84E+00
NEDD9	NEDD9	4.77E-10	4.24E+00	5.96E-07	-2.53E+00
AXL	AXL	1.24E-09	-2.66E+00	1.44E-06	2.29E+00
COL12A1	COL12A1	1.02E-08	3.13E+01	4.09E-11	-6.65E+00
INSR	INSR	2.39E-08	4.89E+00	4.02E-04	-2.33E+00
HMOX1	HMOX1	3.66E-08	-3.67E+00	1.93E-02	2.14E+00
HNRNPA1P7	HNRNPA1P7	3.88E-08	-2.19E+00	1.77E-06	2.04E+00
CFH	CFH	4.73E-08	2.61E+00	1.65E-08	-2.73E+00
PTGFRN	PTGFRN	7.18E-08	5.35E+00	6.59E-07	-3.56E+00
BST2	BST2	9.72E-08	2.74E+00	4.73E-05	-2.01E+00
TGFB2	TGFB2	1.15E-07	1.10E+01	3.23E-12	-4.32E+00
CLDN11	CLDN11	3.48E-07	-2.01E+00	4.36E-08	2.09E+00
TCIM	TCIM	5.67E-07	4.04E+00	7.24E-05	-2.73E+00
DEPP1	DEPP1	6.11E-07	-2.24E+00	8.48E-24	3.98E+00
COL1A2	COL1A2	6.56E-07	2.71E+00	5.93E-08	-2.88E+00
SORBS2	SORBS2	1.93E-06	3.20E+00	1.41E-04	-2.40E+00
TPM2	TPM2	1.58E-05	-3.32E+00	2.36E-07	3.90E+00
ADGRF5	ADGRF5	2.02E-05	3.58E+00	8.57E-03	-2.07E+00
ST6GAL1	ST6GAL1	2.49E-05	-3.12E+00	2.55E-05	3.14E+00
RAPGEF5	RAPGEF5	3.58E-05	2.98E+00	6.14E-05	-2.91E+00
NAMPT	NAMPT	3.93E-05	-2.96E+00	4.63E-02	2.06E+00
PIEZO2	PIEZO2	4.26E-05	-2.67E+00	1.32E-11	4.12E+00
PTGR1	PTGR1	4.30E-05	-2.67E+00	1.15E-02	2.09E+00

TGFBI	TGFBI	7.34E-05	7.42E+00	8.03E-03	-2.92E+00
OAS2	OAS2	9.41E-05	3.31E+00	4.38E-03	-2.19E+00
A2M	A2M	9.64E-05	1.27E+01	9.85E-04	-9.39E+00
SEMA3G	SEMA3G	1.93E-04	1.35E+01	2.65E-04	-4.10E+00
VWA1	VWA1	2.72E-04	1.23E+01	1.16E-04	-8.12E+00
GIMAP8	GIMAP8	2.74E-04	-6.90E+00	1.29E-04	7.50E+00
PIM3	PIM3	3.07E-04	-2.08E+00	2.14E-15	3.64E+00
BCYRN1	BCYRN1	4.49E-04	-2.30E+00	8.68E-03	2.03E+00
SNHG5	SNHG5	4.62E-04	-2.19E+00	7.15E-05	2.34E+00
RESF1	RESF1	6.41E-04	-2.18E+00	2.13E-04	2.29E+00
GJA5	GJA5	6.69E-04	1.99E+01	3.74E-05	-1.28E+01
UNC5B	UNC5B	1.18E-03	6.81E+00	1.83E-02	-3.16E+00
THBD	THBD	1.44E-03	-2.08E+00	2.42E-04	2.24E+00
PTGS1	PTGS1	1.44E-03	-5.29E+00	2.31E-05	7.58E+00
MYO1B	MYO1B	1.46E-03	2.51E+00	1.38E-02	-2.08E+00
DDR2	DDR2	1.73E-03	-3.57E+00	2.00E-02	2.95E+00
ALDH1A1	ALDH1A1	1.88E-03	-2.04E+00	3.79E-11	3.35E+00
UCHL1	UCHL1	2.16E-03	-1.39E+01	4.85E-04	1.80E+01
GIMAP7	GIMAP7	2.25E-03	-5.81E+00	1.53E-03	6.09E+00
COL13A1	COL13A1	2.36E-03	-2.87E+00	3.53E-04	3.22E+00
SCARA3	SCARA3	2.95E-03	-2.55E+00	2.96E-03	2.56E+00
TBX1	TBX1	3.36E-03	1.10E+01	7.16E-04	-7.11E+00
PLAT	PLAT	3.57E-03	-2.86E+00	3.46E-05	3.74E+00
CCDC71L	CCDC71L	6.34E-03	-2.16E+00	4.59E-04	2.46E+00
CNTNAP3B	CNTNAP3B	6.34E-03	-2.39E+00	7.47E-07	3.58E+00
SNORD3B-1	SNORD3B-1	9.49E-03	-6.26E+00	4.17E-03	7.00E+00
DAPK1	DAPK1	1.36E-02	-1.03E+01	2.51E-03	1.43E+01
PALLD	PALLD	1.55E-02	3.49E+00	1.35E-02	-3.12E+00
GJA4	GJA4	1.83E-02	3.30E+00	1.37E-02	-3.64E+00
ATP8A1	ATP8A1	1.90E-02	7.00E+00	2.45E-02	-5.33E+00
TGFA	TGFA	1.96E-02	1.01E+01	1.03E-02	-4.36E+00
PCDH10	PCDH10	2.01E-02	-8.33E+00	9.35E-04	1.43E+01
COLEC12	COLEC12	2.70E-02	-3.21E+00	3.50E-06	6.32E+00
PDE3A	PDE3A	3.50E-02	-2.77E+00	3.08E-02	2.82E+00
FAT1	FAT1	3.88E-02	2.69E+01	1.63E-03	-1.91E+01
PIK3R3	PIK3R3	4.06E-02	2.23E+00	3.79E-03	-2.83E+00
LTBP1	LTBP1	4.21E-02	-2.14E+00	7.49E-04	2.76E+00
